# The Development of CK2 Inhibitors: From Traditional Pharmacology to in Silico Rational Drug Design

**DOI:** 10.3390/ph10010026

**Published:** 2017-02-20

**Authors:** Giorgio Cozza

**Affiliations:** Department of Molecular Medicine, University of Padova, 35131 Padova, Italy; giorgio.cozza@unipd.it; Tel.: +39-049-827-6154

**Keywords:** CK2, inhibitors, structure based drug design, ligand based drug design, cancer, hit optimization

## Abstract

Casein kinase II (CK2) is an ubiquitous and pleiotropic serine/threonine protein kinase able to phosphorylate hundreds of substrates. Being implicated in several human diseases, from neurodegeneration to cancer, the biological roles of CK2 have been intensively studied. Upregulation of CK2 has been shown to be critical to tumor progression, making this kinase an attractive target for cancer therapy. Several CK2 inhibitors have been developed so far, the first being discovered by “trial and error testing”. In the last decade, the development of in silico rational drug design has prompted the discovery, de novo design and optimization of several CK2 inhibitors, active in the low nanomolar range. The screening of big chemical libraries and the optimization of hit compounds by Structure Based Drug Design (SBDD) provide telling examples of a fruitful application of rational drug design to the development of CK2 inhibitors. Ligand Based Drug Design (LBDD) models have been also applied to CK2 drug discovery, however they were mainly focused on methodology improvements rather than being critical for de novo design and optimization. This manuscript provides detailed description of in silico methodologies whose applications to the design and development of CK2 inhibitors proved successful and promising.

## 1. Introduction

CK2 (formerly called Casein Kinase II), was first detected, together with CK1, as early as in 1954 [1]. The conventional term “casein kinase” originally denoted a group of unrelated ser/thr protein kinases able to phosphorylate casein. Only one of these, Fam20C/G-CK (Golgi, or “genuine”, casein kinase) is a bona fide casein kinase in vivo, while CK2 and CK1 share the ability to phosphorylate casein only in vitro. CK2 is an ubiquitous, highly pleiotropic and constitutively active enzyme, responsible for the generation of a significant proportion of the human phosphoproteome [2,3]. CK2 is active as catalytic subunit alone (α and α’) or as tetrameric holoenzyme composed by two catalytic and two regulatory (β) subunits. The α subunit displays the common structural features of all the other member of the human kinome; to note that, unlike many other protein kinases, CK2 displays a constitutively active state, as its activation loop is frozen in an open and active conformation, independently of phosphorylation events [4,5]. CK2 has been linked to a number of human diseases, such as cancer [6], but also cardiac hypertrophy [7], multiple sclerosis [8], virus infections [9,10,11] and cystic fibrosis [12,13,14]. For this reason CK2 is intensively studied as a therapeutic target, especially in the treatment of cancer, and one CK2 inhibitor (CX-4945) is currently in Phase II clinical trials [15,16,17]. 

**Figure 1:**
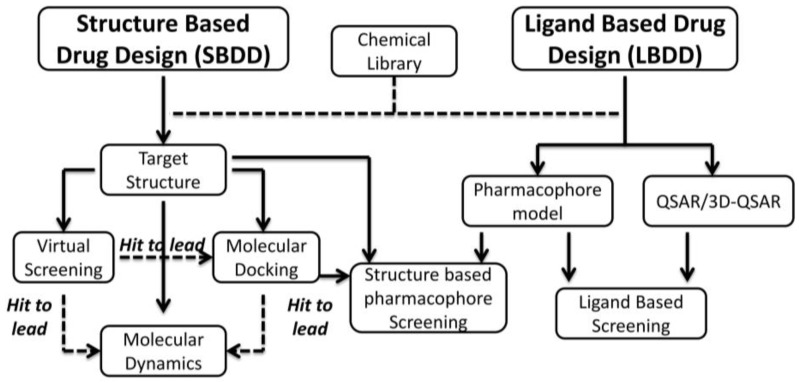
Schematic representation of in silico rational drug design techniques.

Several molecules, belonging to different chemical classes, have shown to inhibit CK2 during the last 20 years. Most of them have been isolated by traditional drug discovery methods, which rely on “trial and error testing” of molecules against the isolated enzyme (recombinant or purified from tissues). More recently many compounds have been discovered and optimized through rational drug design approaches, and in particular by computer aided drug design, combined with in vitro and in cell methodologies supported by crystallographic analysis. Two main groups of techniques should be mentioned, namely structure based and ligand based drug design approaches. Structure-Based Drug Design (SBDD) exploits the three dimensional structure of the biological target, obtained from X-ray crystallography or nuclear magnetic resonance (NMR) spectroscopy, more rarely through Homology modeling (Figure 1). The final goal of this approach is to predict whether chemical compounds are able to interact with a biological target and its affinity. The binding conformation of small molecules into their target, their intermolecular interactions and the structural changes of the drug/target complexes can be estimated through molecular mechanics and molecular dynamics. On the contrary Ligand-Based Drug Design (LBDD) exploits the knowledge of compounds able to interact with the biological target in order to identify a set of chemical features ensuring the molecules activities. This model can be used to design new potent drug-like entities; the pharmacophore approach and quantitative structure-activity relationship (QSAR) are the most used ligand-based methods (Figure 1).

## 2. Structure and Biological Roles of CK2

**Figure 2:**
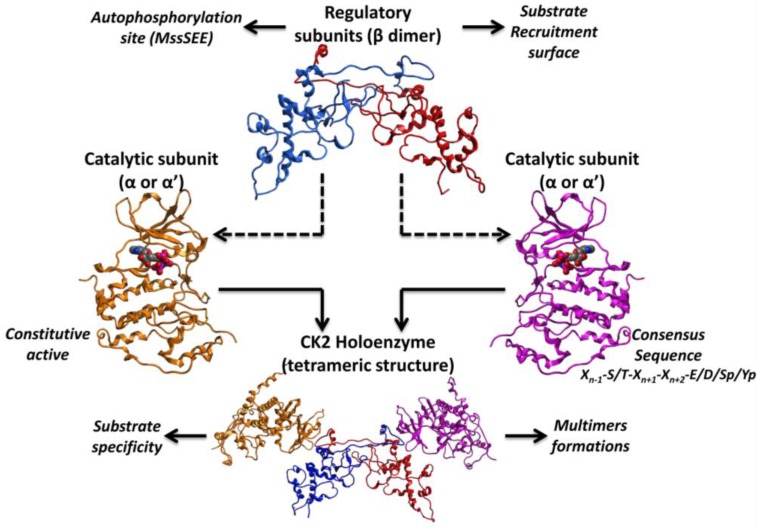
Representation of CK2 structure; catalytic subunits (α and α’), regulatory β dimer and the holoenzyme structure are highlighted.

The first crystal structure of human CK2 holoenzyme (Protein Data Bank (PDB) code: 1JWH) revealed a stable tetramer composed by two catalytic subunit (α and α’), belonging to the CMGC subfamily of the human kinome, and two regulatory β subunit (Figure 2). The two catalytic subunits differ only at the C-terminal domain, while sharing with all the other protein kinases, the main structural features (e.g., the P-loop or glycine-rich loop, the catalytic loop, the activation loop and the substrate binding site). Intriguingly, CK2 is considered a constitutive active enzyme, a rare property among protein kinases; this peculiarity is driven by its N-terminal domain, which is able to form a number of stable interactions with the activation loop [4], whose conformational changes are responsible for the active or non-active state of protein kinases. Normally, the activation loop of active conformer is triggered by single or multiple phosphorylation events. In the particular case of CK2 the N-terminal domain is able to block the activation loop in an open and full active conformation, by its own [4]. CK2 is an acidophilic kinase accepting substrates with the consensus sequence X_n−1_-S/T-X_n+1_-X_n+2_-E/D/Sp/Yp (Figure 2). Even if an acidic residue at position +3 should be sufficient for being a CK2 substrate [18,19], at least five residues have been identified on average around its phosphorylable sites [2]. In fact, the CK2 substrate binding site is characterized by an unique amount of basic residues, in particular the basic stretch of the α-helix C (Lys74-Arg80) has been recognized to be important for substrate recognition, together with the amino acidic triplet, namely Arg191, Arg195 and Lys198 [20]. On the other hand, the mechanism by which the β subunit is able to regulate the activity of the catalytic subunit is still not clarified; however it is known that the β subunit can provide a recruitment surface for substrates, up- (e.g., HIV-rev [21] or eif2β [22]) or down-regulating (e.g., calmodulin [23]) their phosphorylation (Figure 2). Moreover, the autophosphorylation of the β subunit at the N-terminal domain MssSEE (Ser 2 and Ser 3) [24,25] has been suggested to be linked to the formation of multimers based on CK2 tetrameric units, which could play a role in the regulation of CK2 activity [26] (Figure 2).

Protein kinase CK2 is present in many cellular compartments, where it is able to phosphorylate hundreds of substrates. For this reason it has been linked to several cellular processes, being implicated in cell cycle, transcriptional control, neuronal function and response to cellular stress [27,28]. The role of CK2 in many pathologies is well known [27,28]; in particular it is implicated in cardiovascular pathology (hypoxia [29,30,31,32], atherosclerosis [33,34], cardiomyocyte hypertrophy [7]), neurodegeneration (Parkinson’s [35,36,37] and Alzheimer’s diseases [38,39,40,41]), inflammation (glomerulonephritis [42], experimental autoimmune encephalomyelitis, systemic lupus erythematosus [8,43,44], multiple sclerosis [8]), muscle diseases (cardiomyocyte hypertrophy [45,46]) as well as virus and parasite infections [9,10,11]. Moreover a role of CK2 in Cystic Fibrosis (CFTR) has been recently proposed [12,13,14,47]. The pathology where the role of CK2 is best documented and studied is cancer, where this kinase is almost invariably upregulated [6]; recent studies have clearly demonstrated that abnormally elevated CK2 level is required for tumor progression, due to its role in the regulation of almost all the processes essential for cancer development with special reference to the suppression of apoptosis [48]. This dependency of cancer cells to higher level of CK2 in comparison to normal cells, is called addiction, and provides a crucial argument for the development of selective CK2 inhibitors in cancer therapy [48]. 

**Figure 3:**
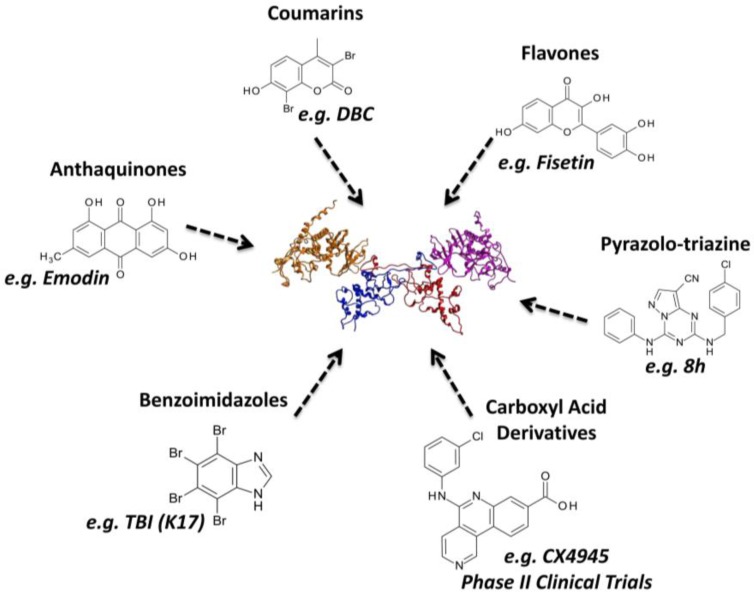
Schematic summary of the most representative families of CK2 inhibitors.

A huge number of CK2 inhibitors are available, most of them belonging to the class I of kinase inhibitors, i.e., compounds able to directly target the ATP-binding site. Benzoimidazoles, anthraquinones, flavonoids, coumarins, and pyrazolotriazines are the most represented families of CK2 inhibitors [27,28] (Figure 3). Many members of these chemical classes were initially found by traditional pharmacology, exploiting trial-and error-testing against the isolated enzyme. With the increasing knowledge of CK2 structure, alone or in complex with different inhibitors, together with the circulation of novel, fast and optimized protocols for computer aided drug design, the rationalization of structural modification became of particular relevance in the design of CK2 inhibitors.

## 3. Rational Drug Design of CK2 Inhibitors: Structure Based Drug Design

The CK2 structure has been intensively studied through X-ray crystallography and homology modeling techniques. Currently a long and growing list of crystal structures is available from the Protein Data Bank (PDB); in particular, as summarized in the Supplementary (Table S1), a consistent group of structures is represented by the catalytic subunit α alone, both in its apo form or in complex with ATP analogs or inhibitors. On the contrary, only a couple of structure of the α’ (PDB codes: 3E2B, 3OFM) and of the entire tetramer are present in PDB (PDB codes: 1JWH, 4NH1, 4MD7, 4MD8, 4MD9, 4DGL). Noteworthy, among the species crystallized, CK2 from *Zea mays* was the most commonly deposited till 2010 (28/40); At a later time, human CK2 has represented the first choice for crystallization (63/75). This abundance of CK2 structures, represents an outstanding resource for in silico drug design and in particular structure based drug design. In fact, these structures provide: (a) high resolution representations of the active site of CK2, useful to design and optimize novel drug-candidates; (b) detailed information about the interactions between CK2 and its inhibitors, which have been demonstrated of particular significance in the training of in silico protocols and scoring functions. The most commonly used SBDD approaches are represented by virtual screening, molecular docking and molecular dynamics (Figure 1). Virtual screening and in particular structure based virtual screening is able to evaluate large libraries of compounds by directly docking the candidates against a structure of interest. Fast molecular docking algorithms are a central part of the procedure together with scoring function protocols able to extract the most promising molecules from the database of millions of compounds. To note that, despite the large number of compounds screened in silico, only a few of them (top selection) will be actually tested and even fewer will be able to achieve a reasonable affinity to the target molecule (see Section 3.2). More accurate molecular docking approaches are exploited in the optimization phase of hit compounds coming from in silico or in vitro screenings, better when combined with crystallographic data. Molecular dynamics simulations have been introduced at a later stage of in silico drug design, with aims of confirm the stability of ligand/target complexes generated from docking studies, and to estimate the free energy of binding between small molecules and their biological targets. 

### 3.1. Protein and Ligand Preparation

SBDD approaches require on one side an accurate 3D structure of the target of intent and on the other a small set or a large library of compounds correctly prepared for in silico calculations. The preparation of the target structure generally starts with the addition of hydrogen atoms to the available 3D system [49,50]; this is particularly the case when crystallographic information is used to describe the biological target, while is not necessary in the case of NMR or homology models. Hydrogen atoms are consequently minimized to avoid contacts, keeping the heavy atoms fixed at their original positions [49,50]. This step can be performed using different types of Force Fields, which represent a set of parameters used to describe atoms and molecules properties (atom types, charges) and to calculate the potential energy of a system. The Force Fields commonly used during protein preparation and during SBDD calculations are generally based on molecular mechanics equations (e.g., Amber [51], CHARMM [52], MMFF [53], OPLS [54]) even if some examples of quantum mechanics or hybrid Force Fields are also available. Unwanted molecules (e.g., ions, ligands, water molecules) are generally excluded in the preparation process, but, in special cases, some of them are considered constitutive of the tridimensional system studied [49,50]. For example during CK2 protein structure preparation, a constitutive water molecule located in the ATP binding site is often maintained in all the in silico calculations [50,55]. Moreover, the selection of the most suitable CK2 crystal structure(s) for SBDD experiments strictly depends on several factors: first of all the SBDD technique applied, the in silico protocols involved, the crystal structure conformations, resolutions etc. In other words the target structure selection must be performed dependently to the computational problem addressed. However, based on the public materials available for SBDD studies on CK2, some general observations can be made. Even if crystal structures of both CK2 α and the tetramer are available, virtual screening and molecular docking procedures are generally based on the structures of the isolated catalytic subunits, because of a better crystallographic resolution and the presence of several inhibitor co-crystallized, missed, instead, in the crystal structures of the tetramer. To note that, even if recently several crystal structures of human CK2 α are available, earlier the in silico analysis on CK2 were exclusively based on *Zea mays* crystal structures. This lack in human CK2 tridimensional information was overtaken by building the human CK2 model through homology modeling technique, exploiting the high similarity between the ATP binding sites of *Zea mays* and *Homo sapiens* CK2 (>98%) [56]. Nowadays crystal structures of the human catalytic subunit are generally selected for virtual screening purpose; this can be done by testing the available protocols to isolate the most suitable structure(s) where the in silico algorithm is able to efficiently reproduce the compounds crystallographic pose. However, *Zea mays* structures remain important to retrieve the binding motif of several known inhibitors of CK2. In some cases, to address specific computational problems, the crystal structure of CK2 tetramer has been also considered. This is the case, for examples of protein-protein docking studies [22,57] or molecular dynamics simulation experiments [5]. 

On the other side, to optimize in silico processes, special care must be taken also to the preparation of chemical libraries; this event is primarily a manual process which starts from the direct building of 3D structure of molecules or from the 2D into 3D conversion of entire database of compounds [49,50]. Protonation states, tautomers and stereochemistry must be taken into account for all the molecules of the chemical library, as well as any desired geometric restraints (distances, angles, dihedrals), if necessary. Finally, partial charges are calculated and applied to the molecules together with an energy minimization protocol with a suitable force field [49,50]. At the end of the preparation process a database of potential candidates is ready to be tested in silico against a biological target. To note that the chemical library is commonly “contaminated” by compounds with known activity against the target of interest. This is of particular relevance during in silico screening campaigns, providing on one side an internal evaluation of the computational approach, on the other an idea of how a scoring function predicts the novel candidates activities in comparison to known ligands [49,50]. 

### 3.2. Virtual Screening Approach

**Figure 4:**
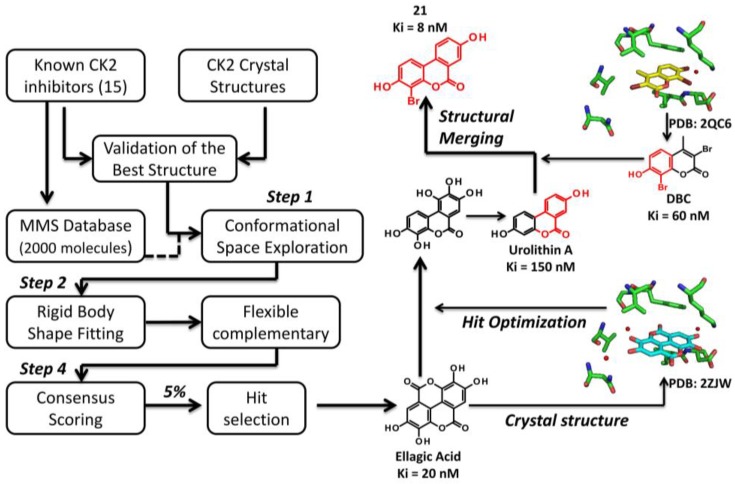
Schematic representation of the structure based virtual screening procedure leading to the discovery of ellagic acid (**left**); on the (**right**) hit optimization of ellagic acid. Inhibition constant (Ki) and PDB (Protein Data Bank) codes have been reported.

Structural-based virtual screening approaches (also defined as high-throughput docking) exploit large libraries of compounds to identify those structures which are able to bind a biological target. The aim of this computational technique is not necessarily linked to the number of hits found during the process; on the contrary, the identification of novel interesting scaffolds is clearly preferable. For this reason two important and connected steps of virtual screening approach must be mentioned: the search algorithm and the scoring function (Figure 4) [58,59]. The search algorithm is able to explore all the possible orientations and conformations of a small molecule within a target binding site. Most of the protocols explore the conformational space of flexible ligands, while the protein structure remains fixed; each final ligand conformation docked inside the protein target is called “pose”. Several search algorithms are available based on different principles: genetic algorithm (Autodock [60], Gold [61], MOE [62]), geometric matching (DOCK [63]), exhaustive search (Glide [64], FRED [65], eHits [66]), incremental search (FlexX [67]). However, defining the best search protocol for virtual screening in general, is not that easy; an evaluation of the available protocols for every case studied, instead, is a mandatory step to identify the best solution for the virtual screening procedure. Since search algorithms are potentially able to generate a huge number of conformations and poses, a procedure suitable to evaluate favorable and not favorable protein/inhibitor complex and to rank one ligand relative to another is required. This procedure is based on scoring function [58,59], which represent an approximate mathematical method used to predict and evaluate the strength of non-covalent interactions between small molecules and target proteins. During virtual screening, it is quite common to use more than one scoring function for the evaluation of the best candidates [49,50]; this procedure is known as “consensus scoring” and it is performed by combining different type of scoring functions in an intersection-based consensus approach. The main advantage of this method is to reduce the numbers of false positives identified by individual scoring functions and to increase the ability to discriminate between active and inactive ligands [49,50] (Figure 4).

#### 3.2.1. Virtual Screening Example 1: The Discovery of Ellagic Acid

A very first example of a structure based drug design technique used to identify novel inhibitors of CK2, was provided by the virtual screening process leading to the identification of the tannin derivative ellagic acid as the most potent CK2 inhibitor at that time [50] (Figure 4). Starting from a relatively small database of natural compounds (2000 molecules), enriched with 15 known CK2 inhibitors to calibrate the high-throughput screening protocol, the authors have set up a consensus screening procedure involving four principal steps (Figure 4). First of all the library was processed with OMEGA, adding hydrogen atoms/Gasteiger partial charges and generating the conformers for the second phase: the rigid body shape fitting. During this step FRED generates a pool of different rigid body poses able to interact with CK2 binding site without generating clashes; this poses were scored and ranked with a Gaussian shape function. Compounds, selected by FRED, that potentially fit CK2 catalytic cleft were subjected to a flexbile docking step, using three different program, namely MOE, Glide and Gold (third step). In the fourth and last phase, the poses generated from the docking procedure were ranked by five scoring function (MOE-Score, GlideScore, GoldScore, ChemScore and Xscore) [50]. A cut-off value for the top 5% compounds ranked by all possible combinations of flexible-docking/scoring functions was selected (consensus docking and scoring), prioritizing 73 molecules for biochemical evaluation [50] (Figure 4). Ellagic acid was selected, among others, as the best compound, resulting the most potent ATP-competitive inhibitor isolated at that time (Ki value of 0.020 μM) [50]. From a small selectivity panel, including 12 representative protein kinases, ellagic acid resulted to be quite selective [50]; this selectivity profile was recently enlarged by testing the residual activity of 70 protein kinases treated with 10μM ellagic acid (Table 1). At this concentration, ellagic acid is able to inhibit, more than 50%, the activity of 21 protein kinases, however only 9 are remarkably affected (residual activity <25%). Beside CK2 (13%), p38-regulated/ activated kinase (PRAK) is the most inhibited (3%) followed by Sphingosine kinase 1 (SPK1) and dual specificity tyrosine-phosphorylation-regulated kinase 3 (DYRK3) (9%), DYRK2 (12%), p21-activated kinase 4 (PAK4) (14%), maternal embryonic leucine zipper kinase 2 (MELK2) (17%), BR serine/threonine-protein kinase 2 (BRSK2) (18%) and Pim-3 proto-oncogene, serine/threonine kinase (PIM3) (21%). The moderate promiscuity of ellagic acid is not so rare among drug candidates from natural sources and could represent the driving force of their activity against diseases like cancer. Indeed, a cytotoxicity profile demonstrated that ellagic acid inhibited the growth of leukemic cells SUDHL1 and FEPD with a DC_50_ (Death Concentration 50) around 30–50 μM after 48-h exposure to the inhibitor [50].

#### 3.2.2. Virtual Screening Example 2: The Discovery of Quinalizarin

**Figure 5:**
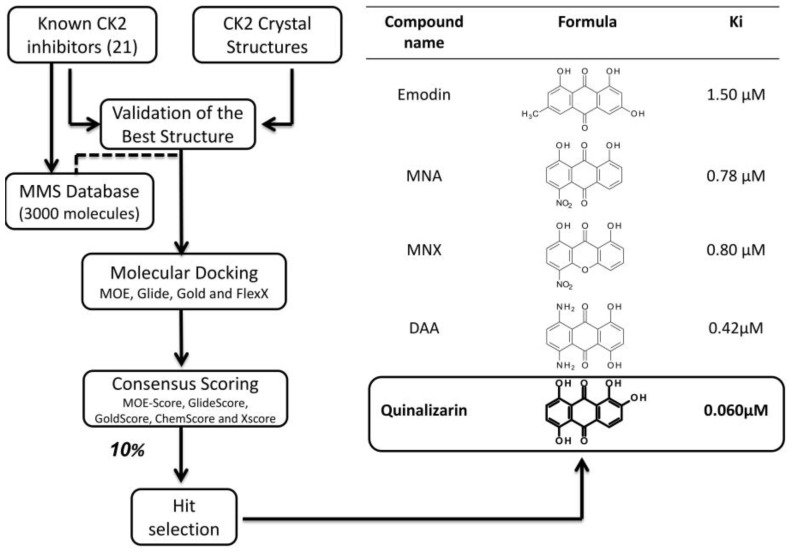
Schematic representation of the structure based virtual screening procedure leading to the discovery of quinalizarin (**left**); whose activity is shown in comparison with the previous discovered anthraquinone inhibitors (**right**).

An implementation of the virtual screening procedure adopted in the case of ellagic acid, led to the identification of quinalizarin as the best anthraquinone inhibitor of CK2 [49]. Starting from an enlarged molecular database (3000 molecules), implemented with 21 known CK2 inhibitors for the calibration phase, a flexible ligand-docking step with four different programs (MOE, Glide, Gold and FlexX) was performed in combination with the five scoring functions described above (cut-off for final selection: 10%) [49]. Quinalizarin, prioritized for biochemical evaluation, resulted to be an ATP competitive inhibitor with a Ki value of 0.052 μM (Figure 5), definitely lower as compared to other anthraquinone derivatives previously identified through traditional pharmacology, namely emodin (6-methyl-1,3,8-trihydroxyanthraquinone, Ki = 1.5 μM) [56], MNA (1,8-dihydroxy-4-nitro- anthracene-9,10-dione, Ki = 0.78 μM), MNX (1,8-dihydroxy-4-nitroxanthen-9-one, Ki = 0.80 μM), DAA (1,4-dihydroxy-5,8-diaminoanthracene-9,10-dione, Ki = 0.42 μM) [69]. Moreover, the first selectivity evaluation of quinalizarin against a panel of 70 protein kinases disclosed a promising specificity for CK2 [49], confirmed by a second assay against an enlarged panel of 140 protein kinases [70]. In particular, while CK2 residual activity, after treatment with 1 μM quinalizarin, resulted to be only 10%, none of the other 140 protein kinases displays a residual activity less than 50%, 132 of them being almost unaffected [70]. Several crystal structures of quinalizarin have been solved firstly by co-crystallization with *Zea mays* CK2 (PDB code: 3FL5 [10]), later with human CK2 (PDB codes: 3Q9Z and 3Q9Y [29]). These structural resources are exploited to disclose on one side the molecular features underlying quinalizarin selectivity and, on the other, its preference for CK2 holoenzyme over the CK2 α alone [70]. To note that, quinalizarin is readily cell permeable and very effective as pro-apoptotic agent, having been successfully used in many studies concerning CK2 physiological and pathological roles, as well to disclose proteomics perturbations caused by CK2 down regulation [71].

#### 3.2.3. Virtual Screening: Other Examples

One of the first examples of a successful virtual screening procedure applied to the identification of CK2 inhibitors was performed by Novartis Pharma: a fast high-throughput screening protocol (DOCK software), combined with a pharmacophore filter, was used to screen a database of 400,000 compounds. A dozen compounds were retrieved from the in silico procedure; of particular interest was a quinazoline derivative (compound **4**, 5-oxo-5,6-dihydroindolo- [1,2-a]quinazolin-7-yl, later named IQA), displaying an IC_50_ (half maximal inhibitory concentration) of 0.080 μM against CK2 and a good selectivity towards a small panel of 21 representative protein kinases [72]. Hereafter, IQA was profiled against 44 kinases, confirming its selectivity and prioritized for in cell assays, showing remarkable efficacy in Jurkat cells [73]. The crystal structure of IQA and *Zea mays* CK2 was solved (PDB code: 1OM1), paving the way for further optimization of this promising scaffold [73].

Another example of a virtual screening application exploited the chemical structure of CX4945, the only CK2 inhibitor in clinical trials; a two-steps shape based virtual screening approach was used to retrieve novel compounds able to inhibit CK2 [74]. Firstly, a shape-based model derived from CX-4945 was built, and used to screen a database of molecules with the OMEGA/ROCS software, leading to the identification of one quinazoline derivative (SHP01, IC_50_ = 4.23 μM), whose interactions with CK2 active site were later clarified through molecular docking (GOLD software). Based on SHP01 scaffold, a second shape based model was built and a new set of compounds from the screening analysis have been prioritized for molecular docking and biochemical evaluation. The three best compounds SHP19, SHP26 and SHP27 (IC_50_ = 0.46 μM, 0.69 μM and 0.55 μM respectively) were also assayed for their cytotoxicity in several cell lines, displaying a DC_50_ values around 30 μM [74]. Although this strategy proved promising for the optimization of SHP01 inhibitory activity, it was not able to retrieve compounds as effective as CX4945. Moreover, the selectivity of the hit molecules identified in the virtual screening procedure remained undetermined, as well as the real contribution of CK2 to the cytotoxic effect of these chemical entities.

Finally an in silico screening application, called Cross-Docking-based Virtual Screening, was used to explore a database of over 300,000 compounds previously resized to around 80,000 using specific druglike property filter [75]. Ninety six compounds were retrieved from the screening procedure and submitted to a toxicity prediction procedure; only seven of them were assayed in vitro, compound **g** resulting to be the best inhibitor, with an activity in the micromolar range [75].

### 3.3. Molecular Docking, Molecular Dynamics and Hit Optimization (Hit to Lead)

Compounds selected from a virtual screening procedure (for example ellagic acid or quinalizarin) are generally defined as hits and evaluated by means of binding affinity for their biological target as well as for their selectivity toward unwanted off-targets. After their efficacy is validated in cell environment, to assess their cell permeability and ability to perturb biological functions. Co-crystallization of hits and the target protein is a crucial step in hit evaluation, since it provides significant information about the molecular interactions and may confirm or not the poses obtained from the in silico high-throughput screening procedure. Hit to lead represents a stage in early drug design where hits undergo a rational optimization of their chemical scaffold to improve, among others, chemical and metabolic stability, affinity and selectivity towards the biological target, as well as efficacy in cellular assays. Hit optimization is generally performed by exploiting optimized docking protocols as well as molecular dynamics simulations; these approaches are especially useful when coupled with crystallographic information about hit/target complexes. The design of novel compounds starting from existing hits can be evaluated through molecular docking which is able to predict the binding motif of optimized molecules and their affinity towards the biological target, using different scoring functions. The comparison between hit compounds binding affinity and the ones of novel designed candidates, helps to direct new synthesis of optimized molecules. On the other hand, molecular docking can be useful to determinate the Structure Activity Relationship (SAR) of a family of compounds variably active against a target. In fact the knowledge of the relationship between the chemical or 3D structure of a molecule and its biological activity, is critical to understand which kind of chemical substitutions are expected to improve the activity of hit compounds. Similarly, molecular dynamics simulation can be used to estimate the free binding energy of small ligands to biological macromolecules. In particular molecular dynamics calculations are used to study target/ligand complexes, to rationalize experimental findings and to improve the results of virtual screening and docking. Molecular mechanics (MM) energy coming from molecular dynamics experiments are coupled with PBSA and GBSA (Poisson Boltzmann or generalized Born and surface area continuum salvation) methods to estimate ligand binding affinities to the biological target [76]. To note that many attempts to improve these methodologies have been performed by exploiting for example Quantum Mechanics (QM) approaches. Liner Interaction Energy (LIE) is another method for hit optimization coupled with molecular dynamics simulations: it consists in a semiempirical approach that combines the advantages of Free Energy Perturbation (FEP) and Thermodynamic Integration (TI), by calculating binding free energies of the bound and the free state of ligands [77].

After hit optimization, the newly generated molecules are generally called lead compounds; they are further improved in a lead optimization phase, exploiting the acquisition of Adsorption, Distribution, Metabolism, Excretion, Toxicity (ADMET) properties, till the development of drug candidates ready for in vivo testing. This issue will not be considered in this paper because, although many inhibitors of CK2 have reached in vivo studies, in silico lead optimization of this molecules has not been performed.

#### 3.3.1. Hit to Lead: Ellagic Acid

The optimization of the CK2 inhibitor ellagic acid is a telling example of a hit to lead strategy starting from a compound retrieved by a virtual screening technique. Starting from the crystallographic structures of ellagic acid and the cumarin derivative DBC (3,8-dibromo-7-hydroxy-4-methylchromen-2-one, Ki = 0.06 μM ) in complex with the α-subunit of CK2 (PDB codes: 2ZJW and 2QC6, respectively [78,79]), an analysis of the interactions of the two compounds with CK2 catalytic site was performed (Figure 4). Ellagic acid and DBC display a similar binding mode; however, while ellagic acid is able to interact with both CK2 hinge region and the phosphate group binding area, DBC is able to directly bind only the hinge. To note that in 2008, a LIE model approach was performed on DBC and more than 60 coumarins derivatives, to define and understand the importance of different energy contributions to the binding free energy of this class of compounds [79].

From these observations, a molecular simplification of the ellagic acid scaffold has been proposed leading, among others, to an hydrolysable tannin, urolithin A (IC_50_ = 0.39 μM) [55] (Figure 4). Interestingly, the molecular docking experiments of urolithin A inside CK2 catalytic site, demonstrate that ellagic acid and urolithin A share the same binding motif characterized by the two crucial hydrogen-bonding interactions [55]. This was also confirmed by methylating, individually or together, the hydroxy groups at position 3 and 8, resulting in two molecules with low affinity (compound 9, IC_50_ = 3.5 μM) or completely inactive (compound **10**, IC_50_ > 40 μM) [55]. Moreover, being urolithin A a benzocoumarin derivative, it was suggested to represent a bridging scaffold between ellagic acid and DBC (Figure 4). For this reason, the effect of electron-withdrawing substituents (nitro and bromine), was investigated through molecular docking approach [55]. Among various derivatives proposed in silico and prioritized for chemical synthesis and biochemical evaluation, 4-bromo-3,8-dihydroxy-benzo[c]chromen-6-one (compound **21**, Ki = 0.007 μM) resulted to be even more efficacious than ellagic acid and DBC (Figure 4) [55].

#### 3.3.2. Hit to Lead: Benzimidazole Scaffold

**Figure 6:**
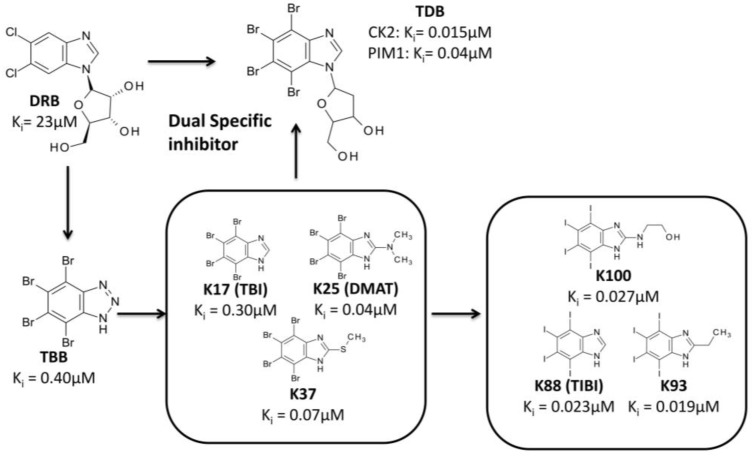
Schematic representation of the optimization of tetrabromobenzimidazoles.

The optimization of chemical entities in drug discovery can be also performed starting from a scaffold recognized as a hit compound, even if not selected by high-throughput screening. One of the first family of compounds identified as inhibitors of CK2 is represented by polyhalogenated benzimidazole derivatives. DRB (5,6-dichloro-1-(β-d-ribofuranosyl)benzimidazole, Ki = 23 μM) [80], TBB (4,5,6,7-tetrabromobenzotriazole, Ki = 0.40 μM) [81], and the first generation of benzimidazole derivatives were discovered by traditional pharmacology (“trial and error testing”) [82]. Several experimental data were collected for this first series of compounds, in particular focusing on the determination of their in vitro (e.g., K25 Ki = 0.04 μM) and in cell potency, their mechanism of action and their selectivity towards different kinases panels, as well as on the co-crystallization of some of them with CK2 catalytic subunit (Figure 6). Such as amount of data was essential to determinate a SAR for this starting group of compounds, and to perform additional modifications of the benzimidazole scaffold, exploiting the crystallographic informations available. First of all, the role of different halogen substitutions (Figure 6) was evaluated through molecular docking analysis combined with biochemical assays, proving that tetraiodined compounds present, on average, IC_50_ one of magnitude lower than the corresponding brominated ones (e.g., K88 Ki = 0.023 μM; K93 = 0.019 μM; K100 Ki = 0.027 μM) [83].

**Figure 7:**
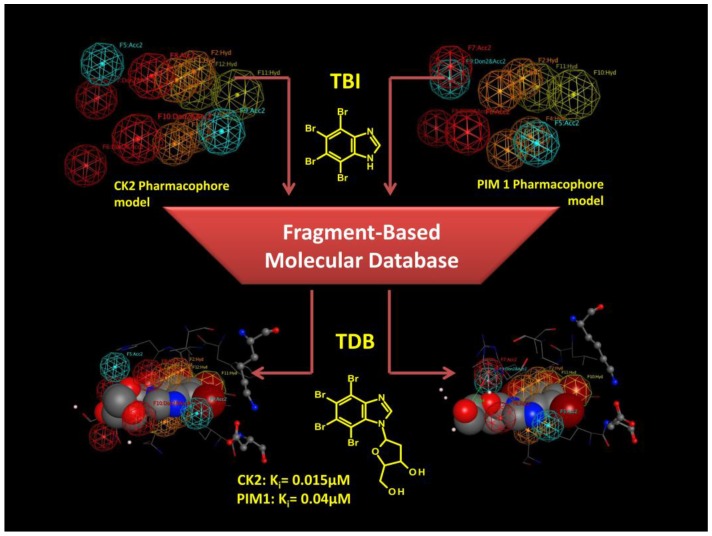
Structure based pharmacophore screening applied for the discovery of TDB (1-β-D-2′-deoxyribofuranosyl-4,5,6,7-tetrabromo-1H-benzimidazole), a CK2 and PIM1 dual inhibitor. Spheres represent chemical features: Red for donor, blue for acceptor, orange and yellow for hydrophobic/aromatic.

Secondly, the benzimidazole scaffold was further optimized to design a molecule able to inhibit CK2 together with other protein kinases implicated in the same human disease [84] (Figure 6 and Figure 7). The goal of such a strategy, called Multi Target Drug Design (MTDD), is to block redundant compensatory pathways in diseases like cancer or neurodegeneration, with consequently increase of efficacy and the reduction of drug resistance. Compounds able to target two or more protein kinases are called Multi Kinase Inhibitors (MKI); several example of MKI are present in literature, some of them have been also approved by FDA, like imatinib (Gleevec) [85] and lapatinib (Tykerb) [86,87]. The crosstalk between CK2 and PIM1 (kinase of the Proviral Integration of Moloney virus) [88] has been recognized in a number of tumors (e.g., prostate cancer, and hematologic malignancies) where they are implicated in the resistance to apoptosis [84]. The design of a specific dual and cell permeable inhibitor of CK2 and PIM1 was obtained through an in silico rational optimization of the benzimidazole scaffold, in particular by exploiting a structure based pharmacophore approach [84] (for details about pharmacophore model see Section 4 ). Even through this method can be used also with apo protein structures, in this case it was used to obtain pharmacophores from CK2 and PIM1 structures in complex with different inhibitors (Table 2). In fact, beside CK2, several co-crystal structures of PIM1 and its inhibitors are also available. Once collected, the structural data from both CK2 and PIM1 were divided into two groups; the first one representing the training set for the pharmacophore selection, the second one representing the test set to validate the pharmacophore model (Table 2). For both CK2 and PIM1, one single structure was chosen to generate the pharmacophore model taking into account all the interactions performed by CK2 and PIM1 ligands into the respective binding site, according to the training set. At the end of the procedure a set of chemical features was retrieved and clusterized into hydrogen donor, hydrogen acceptor, hydrophobic and aromatic features (Figure 7). Only the most recurring features retrieved from the protein ligand interaction pattern, were selected and included in the final generation of the pharmacophore models of CK2 and PIM1. These models were validated by using molecules from the test set; to note that all the inhibitors of CK2 and PIM1 present in the test set have not been used for the pharmacophore model determination, being, however, positively selected by the two final pharmacophore models, in the validation procedure. Couriously the pharmacophore models of CK2 and PIM1 resulted to be geometrically and qualitatively quite similar, being composed of two acceptor features, five hydrogen/aromatic feature, while four donor features were present in CK2 instead of three in PIM1 (Figure 7). Both the validated pharmacophore models were used as 3D queries in database searching. The chemical library represents a family of 350 tetrabromobenzimidazole derivatives built in silico through combinatory chemistry of small chemical fragments (Figure 7). Among others, TDB (1-β-D-2′-deoxyribofuranosyl-4,5,6,7- tetrabromo-1*H*-benzimidazole) resulted to be the most promising molecule retrieved from the screening procedure [84]. As expected TDB showed an ATP competitive inhibition with both CK2 and PIM1 (Ki values, 0.015 and 0.040 μM respectively), and a promising selectivity, being almost ineffective against a panel of 124 protein kinases, at 1 μM concentration [84]. The selectivity of TDB is also confirmed by the values for the Gini coefficient (0.553) and hit rate (0.14), denoting that TDB is one of the most specific inhibitors of CK2. Only two other protein kinase, DYRK1a and CLK2 have been shown to be affected by TDB (IC_50_ = 0.4 μM and 0.02 μM, respectively) [84]. To note that the overexpression of CLK2 is also implicated in many tumors, thus the triple specificity of TDB against CK2, CLK2 and PIM1, should be considered an advantage, rather than a weakness. The specificity of TDB towards CK2 and PIM1 was also demonstrated in living cells, where the compound was able to inhibit the endogenous activity of both protein kinases, and to reduce the phosphorylation level of specific substrates of CK2 and PIM1 (Akt Ser129 and Bad Ser112, respectively) [84]. To note that, the combined efficacy of TDB against CK2 and PIM1 was probably responsible of the remarkable cytotoxicity of the compound against cancer cells (CEM and HeLa) compared to non-tumor (cells CCD34Lu and heK-293t), showing that TDB was even better than CX-4945 in a comparative MTT test on CEM cells [84].

**Figure 8:**
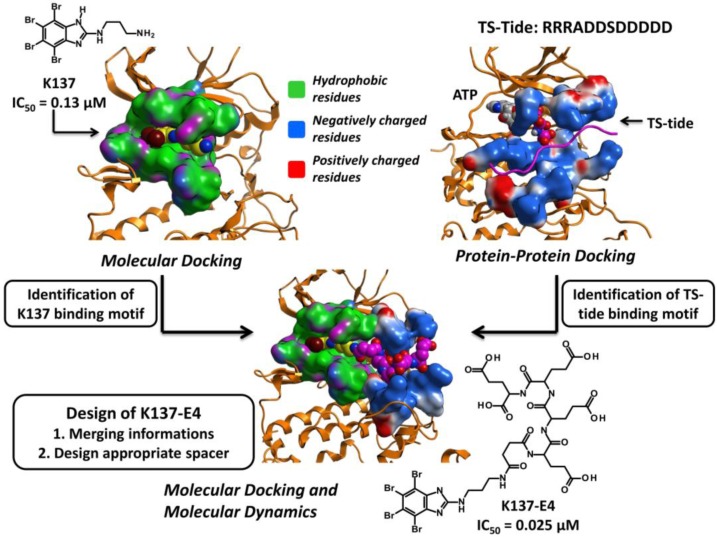
Schematic representation of the rational strategy leading to the design of bisubstrate inhibitors.

Another modification of the benzimidazole scaffold was rationalized starting from one of its ATP competitive derivative K137 (N1-(4,5,6,7-tetrabromo-1*H*-benzimidazol-2-yl)-propane- 1,3-diamine), with the aim to design a new family of bisubstrate inhibitors able to simultaneously interact with the ATP and the phosphoacceptor substrate binding sites [89] (Figure 8). To this purpose the extended knowledge acquired about the interactions between CK2 and its acidic peptide substrates (e.g., TS-Tide, RRRADDSDDDDD), together with the huge amount of crystallographic data for ATP competitive inhibitors, was transferred to a Molecular Dynamics guided Structure Based Docking procedure [89] (Figure 8). First of all the interaction pattern of K137 and of TS-Tide with CK2 ATP and substrate binding site respectively was acquired, through Docking and Molecular Dynamics simulations. In particular K137 binding pose was retrieved through a genetic algorithm based molecular docking (Gold software, genetic algorithm), while the CK2-peptide substrate complex was obtained through a protein-protein docking procedure (PIPER software [90]). 1000 complexes were obtained from protein-protein docking algorithms and clusterized using the pairwise Root Mean Square Deviation (RMSD) into the six largest clusters. The final complex was chosen according to the energy scoring function. Both the docking experiments were submitted to a Molecular Dynamics protocol (NAMD 2.8 [91], 100 ns of NPT, 1 atm, 300 K) to optimize the interactions within the complexes. These data were used to propose a binding motif for K137, to identify the minimum interactions required to allocate the acidic peptide substrate in CK2 substrate binding site, and to design the best chemical spacer to connect the K137 moiety to the acidic peptide. From this procedure, a small group of molecules, was prioritized for biochemical evaluation [89]; the best compound resulted to be K137-E4 (IC_50_ = 0.025 μM) in which K137 was derivatized in position 3 by a chemical spacer connected to a peptidic fragment composed by 4 glutamic acid [89] (Figure 8). From the in silico analysis, K137-E4 was predicted to interact in the ATP binding site through the K137 moiety, and in the substrate binding region with the peptide portion (E4). Mixed competition kinetics and mutational analysis demonstrated in vitro that the compound is indeed able to interact simultaneously with both the ATP and substrate binding sites [89]. In addition to a highter potency as compared to K137 (IC_50_ = 0.13 μM), K137-E4 is more selective: while K137 is able to inhibit 35 out of 140 protein kinases more potently than CK2, K137-E4 is only active against CK2 [89]. This remarkable selectivity is also demonstrated by the value of the hit rate for K137-E4 (0.05) much lower than the one for K137 (0.27) [89].

#### 3.3.3. Hit to Lead: Other Examples

In the absence of crystallographic informations, molecular docking has been widely used to determine the binding motif of CK2 inhibitors. This technique was particularly useful to explain the SAR of several families of compounds, and to design optimized molecules starting from their docking pose. For example, a family of pyrimidine derivatives [92] was recently further optimized, generating a series of novel ATP-directed compounds with variable activities against CK2; one of them NHTP23 (3-(5-phenylthieno [2,3-d]pyrimidin-4-ylamino)benzoic acid) was extremely active (IC_50_ = 0.01 μM) and selective against a small panel of eight protein kinases [93]. Likewise, a novel family of tetrabromobenzotriazole derivatives was studied for their inhibitory activity toward CK2 and rationalized through docking experiments. R-7b (IC_50_ = 0.80μM) was the best hit compound obtained, being however effective in MCF7 adherent cells only at concentration between 50 and 100 μM [94]. To note that molecular docking was also used to clarify the binding mode of the promising peptide inhibitor Pc, able to interfere with the interaction surface between CK2 catalytic (α) and regulatory (β) subunits; this results could pave the way to the development of allosteric inhibitors of CK2 [95].

Many other computational studies have been performed on existing families of CK2 inhibitors. However, most of them have been dedicated to the development of new computational strategies and techniques, to clarify the SAR of existing families of compounds and their interaction with CK2, without leading to novel optimized chemical entities. For example, the tricyclic quinoline compound CX-4945, the only CK2 inhibitor in clinical trials, has been studied in silico, through molecular docking and dynamics, to clarify the role of its acidic portion, responsible for its marked activity [96]. In particular, the presence of non-R2 carboxylate function resulted in a different protein-ligand recognition, leading to unfavourable electrostatic interactions with the ATP binding site of CK2 [96]. Another example is provided by the exploration of interactions between a group of tetrabromobenzimidazole derivatives with CK2, exploiting a QM/MM-PB/SA method [97]. This approach was used to estimate the binding free energies of CK2-inhibitor complexes obtained through QM/MM molecular dynamics. The results demonstrated that the contribution of solvation (PB/SA) is essential to retrieve reliable results, that the hydrophobic contribution represents the driving force for the binding, while the electrostatic interactions are important for the correct orientation of benzimidazole inhibitors in the CK2 active site [97]. Moreover, the in silico methodology suggested modifications able to potentially increase the binding affinity, however no evaluation of these hypothetical molecules is actually available.

## 4. Rational Drug Design of CK2 Inhibitors: Ligand Based Drug Design

The huge number of crystallographic structures available for CK2, have tipped the balance of computational studies in favor of structure based drug design. However, some examples of efficient Ligand-Based Drug Design (LBDD) procedures applied to the development of CK2 inhibitors are also available in literature (discussed below). Instead of being focused on the biological target, like in the case of SBDD, LBDD exploits the knowledge of known active molecules to predict novel chemical entities able to affect the target of interest. To note that this approach, even if can be combined with SBDD techniques, it is born to be independent from the structure of the target, thus can prove extremely helpful to develop novel ligands against targets lacking tridimensional information. A huge amount of CK2 inhibitors (>200) are available in literature, belonging to several chemical class of compounds (e.g., anthraquinone, coumarin, flavone, quinoline) and characterized by a wide range of in vitro activities and selectivity (see specific reviews for detailed informations [27,28]). This structure diversity should be considered an advantage in the application of LBDD approaches for the developing and optimization of CK2 inhibitors. For this purpose, Pharmacophore screening [98] and Quantitative Structure–Activity Relationship (QSAR/3D-QSAR) [99,100] are the most used LBDD, the former mainly focused on the structural geometries and characteristics of known compounds, the latter on the correlation between calculated chemical properties of molecules and their experimentally determined activity.

### 4.1. The Pharmacophore Approach

A pharmacophore can be defined as an ensemble of chemical features, generally consisting of hydrogen bond acceptors/donors, electrostatic features and hydrophobic centroids, necessary to ensure the molecular recognition between a ligand and its biological target. A generated pharmacophore model could identify the minimum chemical features which different ligands should possess to bind a common binding site and can be used in a ligand-based virtual screening to identify new molecules with the same features [98]. It is normally built from a training set of diverse molecules active against the same biological target, whose low energy conformers are generated and superimposed. The 3D alignment of the ligands is used to extract chemical properties for the pharmacophore model, assigning the correct pharmacophoric features [98]. The final model must be validated with a set of known active compounds against the common biological target. It is fairly common for pharmacophore approach to be combined with SBDD techniques when tridimensional information for the biological target is available. For example, results from a ligand-based pharmacophore model are often confirmed through molecular docking before being validated by biochemical evaluation.

#### 4.1.1. The Pharmacophore Approach: Applications

An interesting combination of pharmacophore hypothesis, the Bayesian model (LBDD technique) and molecular docking was recently performed [101]. Bayesian model, a probabilistic classifier based on applying Bayes’ theorem, is one of the most versatile machine learning algorithms. It was used to distinguish active from non-active inhibitors of CK2, by training the protocol with a set 73 active and 29 inactive compounds. On the other side a pharmacophore model was developed using seven active CK2 inhibitors together with two non-active compounds; finally a docking protocol was used to confirm the data obtained from LBDD analysis. Bayesian model, pharmacophore model and molecular docking were sequentially used to filter more than one million molecules; 30 compounds were selected from the in silico analysis and one of them (compound **C1**) resulted to be structurally unrelated to the other known CK2 inhibitor and displayed an IC_50_ of 5.85 μM [101].

### 4.2. Quantitative Structure–Activity Relationship (QSAR)

Quantitative structure-activity relationships (QSAR) can be defined as regression or classification models used in several scientific applications. In computational chemistry and in drug design in general, QSAR have been applied to study the relationship between physicochemical properties of molecules and their biological activities; QSAR aims at building reliable statistical models to predict the activities of novel chemical entities [99,100]. The basic concept of QSAR is that structural properties of molecules are strictly connected with their biological activities; in other words inhibition constants, rate constants and affinities of compounds with their targets are correlated with chemical features like electronic and steric properties, lipophilicity, polarizability. 3D-QSAR has emerged as an optimized methodology for the design of new molecules, by exploiting the three-dimensional properties of known compounds to predict the biological activities of novel chemical entities through chemometric techniques such as Partial Least Squares (PLS), Principal Components Analysis (PCA), Comparative Molecular Field Analysis (CoMFA), Molecular Similarity Indices in a Comparative Analysis (CoMSIA).

#### 4.2.1. The QSAR Approach: Applications

By exploiting a pool of 38 CK2/inhibitors complexes a QSAR model based on Multiple Linear Regression (MLR) was built in combination with a docking protocol (AutoDock) for the generation of energy-based descriptors. After a cross-validation procedure, 20 analogues of ellagic acid were subjected to the QSAR-Docking approach; intriguingly two compounds were predicted in silico to be more potent than ellagic acid, however these results were not validated in a biochemical assays [102]. On the other hand, CoMFA and CoMSIA methodologies, based on several CK2 inhibitors, were used in a 3D-QSAR study, generating a statistically solid model; unfortunately, the in silico analysis was not applied for the discovery of novel compounds, but only suggested plausible substitutions in the development of CK2 inhibitors [103]. CoMFA and CoMSIA descriptors were also applied to study 40 coumarin derivatives combined with molecular docking. The model was successfully validated using five known inhibitors of CK2 belonging to the coumarin family of compounds [104].

## 5. Discussion

CK2 is an ubiquitous and pleiotropic protein kinase, intensively studied for its biological roles and its implication in several human diseases in particular cancer, where its upregulation favours tumor progression. The search of CK2 inhibitors started several years ago and at the beginning it was grounded on “trial and error testing” against the purified enzyme. With the increasing number of crystallographic information about CK2 alone or in complex with the first isolated inhibitors, together with the availability of in silico protocols for the design and development of active ligands, several research groups started to merge the classical biochemical studies with computational methodologies able to rationalize the experimental data. Later on, in silico rational drug design became even more important for the discovery and optimization of CK2 inhibitors in particular exploiting Structure Based Drug Design (SBDD) techniques. The abundance of crystal structure of CK2 proved very helpful for the optimization of virtual screening and molecular docking algorithms, as well as for the building of solid free energy binding models. Several promising compounds have been obtained through virtual screening campaigns, like IQA, ellagic acid and quinalizarin. Moreover, rational hit optimization by means of molecular docking, pharmacophore approaches and molecular dynamics simulations has been shown to be able to retrieve novel potent inhibitors of CK2, with different mechanism of action and specificity.

Special reference needs to be made for Ligand-Based Drug Design (LBDD) techniques; despite the abundance of CK2 inhibitors available in the literature, the success of these approaches is very limited; in fact LBDD techniques were able to suggest new scaffolds, however characterized by poor potency and not further optimized. The general impression is that the development of CK2 inhibitors through LBDD techniques is still in an early phase, probably restrained by the presence of validated and efficacious SBDD protocols. This is confirmed also by the majority of published papers, where LBDD techniques are applied for the design of CK2 inhibitors. In these cases, indeed, calibration, validation and improvement of the methodologies are the main addressed aspects, rather than specific applications for the development of potent and selective inhibitors of CK2.

**Figure 9:**
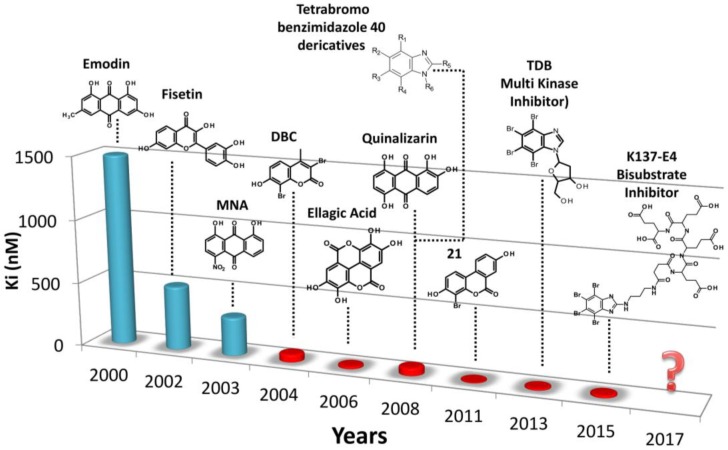
Activity of CK2 inhibitors over time expressed in Ki; blue bars represent the inhibition constant of compounds identified through random screen campaign of chemical compounds (“trial and error testing”), red bars the ones of compounds discovered through in silico rational drug design.

In conclusion, as summarized in Figure 9, the introduction of in silico rational drug design in the discovery and optimization of CK2 inhibitors was of invaluable usefulness to retrieve novel hit compounds by virtual screening and to focalize the synthesis of optimized molecules by molecular docking and dynamics. The combination of in silico techniques with biochemical, crystallographic, and in cell approaches has accelerated the discovery of more active and more selective inhibitors of CK2, as compared to the traditional trials and error testing. Moreover the increasing of computational power based not only on the Central Processing Unit (CPU) but also on the Graphics Processing Unit (GPU), gives a promising perspective on the development of even better computational algorithms for CK2 drug design, involving mechanics calculation in the quantum level based on larger databases of compounds.

## Figures and Tables

**Table 1:** Selectivity profiles of ellagic acid on a 70 kinase panel. Residual Casein kinase II (CK2) activity (determined at 10 μM Ellagic Acid concentration) is expressed as a percentage of the control activity without inhibitor. Activities <25% of control are highlighted in grey. Conditions for the activity assays are described in [68].

Kinase	% Activity	Kinase	% Activity	Kinase	% Activity
PRAK	**3**	PDK1	**66**	Lck	**101**
SRPK1	**9**	PKC zeta	**67**	HIPK3	**102**
DYRK3	**9**	MSK1	**68**	ERK1	**103**
DYRK2	**12**	S6K1	**68**	PRK2	**105**
CK2	**13**	ROCK 2	**68**	JNK3	**106**
PAK4	**14**	PKA	**69**	P38b MAPK	**107**
MELK	**17**	PKBb	**71**	EFK2	**111**
BRSK2	**18**	CSK	**73**	CHK2	**117**
PIM3	**21**	PLK1	**76**	CAMKKa	**118**
DYRK1A	**26**	Src	**76**	SmMLCK	**127**
MAPKAP-K2	**26**	JNK1	**78**		
PAK5	**29**	SGK1	**80**		
CAMKKb	**29**	PKBa	**80**		
GSK3b	**30**	PKCa	**81**		
IKKb	**36**	CHK1	**81**		
MAPKAP-K3	**39**	NEK7	**82**		
PIM1	**40**	PHK	**82**		
PAK6	**42**	CAMK1	**82**		
ERK8	**46**	ERK2	**84**		
AURORA C	**46**	AMPK	**86**		
MARK3	**47**	JNK2	**87**		
PKD1	**48**	HIPK2	**89**		
PLK1	**51**	NEK6	**91**		
RSK2	**51**	MNK2	**94**		
CK1	**58**	p38s MAPK	**98**		
RSK1	**58**	p38a MAPK	**100**		
MKK1	**62**	MNK1	**100**		
AURORA B	**64**	p38g MAPK	**101**		
NEK2a	**65**	MST2	**101**		
PIM2	**65**	CDK2-Cyclin A	**101**		

**Table 2:** List of CK2 and PIM1 crystal structures (PDB codes) used as training and test set for pharmacophore building.

CK2		PIM1	
Training Set	Test Set	Training Set	Test Set
3KXM	3KXN	4ENY	3UIX
3KXH	3KXG	4A7C	3T9I
3PVG	3PWD	3R00	3R01
3NGA	3Q9Y	3R02	3R04
3AMY	3OWK	3XJ1	3XJ2
4DGN	3MB7	3JPV	3DCV
3OWL	3OWJ	3C4E	3BGP
3MB6	3RPS	3BGQ	3BGZ
1ZOH	1ZOG	3UMX	4ENX
1M2R	1M2Q	4ALW	3UMW
2OXD	2OXY	4ALU	4ALV
1OM1	1M2P	4K18	4K1B

## Supplementary Materials

The following are available online at http://www.mdpi.com/1424-8247/10/1/26/s1, Table S1: Summary of all CK2 crystal structures deposited in the Protein Data Bank (PDB).

## References

[1] Burnett G., Kennedy E.P. (1954). The enzymatic phosphorylation of proteins. J. Biol. Chem..

[2] Meggio F., Pinna L.A. (2003). One-thousand-and-one substrates of protein kinase CK2?. FASEB J..

[3] Salvi M., Sarno S., Cesaro L., Nakamura H., Pinna L.A. (2009). Extraordinary pleiotropy of protein kinase CK2 revealed by weblogo phosphoproteome analysis. Biochim. Biophys. Acta.

[4] Sarno S., Ghisellini P., Pinna L.A. (2002). Unique activation mechanism of protein kinase CK2. The n-terminal segment is essential for constitutive activity of the catalytic subunit but not of the holoenzyme. J. Biol. Chem..

[5] Cristiani A., Costa G., Cozza G., Meggio F., Scapozza L., Moro S. (2011). The role of the n-terminal domain in the regulation of the “constitutively active” conformation of protein kinase CK2alpha: Insight from a molecular dynamics investigation. ChemMedChem.

[6] Ortega C.E., Seidner Y., Dominguez I. (2014). Mining CK2 in cancer. PLoS ONE.

[7] Murtaza I., Wang H.X., Feng X., Alenina N., Bader M., Prabhakar B.S., Li P.F. (2008). Down-regulation of catalase and oxidative modification of protein kinase CK2 lead to the failure of apoptosis repressor with caspase recruitment domain to inhibit cardiomyocyte hypertrophy. J. Biol. Chem..

[8] Axtell R.C., Xu L., Barnum S.R., Raman C. (2006). Cd5-CK2 binding/activation-deficient mice are resistant to experimental autoimmune encephalomyelitis: Protection is associated with diminished populations of il-17-expressing t cells in the central nervous system. J. Immunol..

[9] Ivanov K.I., Puustinen P., Gabrenaite R., Vihinen H., Ronnstrand L., Valmu L., Kalkkinen N., Makinen K. (2003). Phosphorylation of the potyvirus capsid protein by protein kinase CK2 and its relevance for virus infection. Plant Cell.

[10] Foka P., Dimitriadis A., Kyratzopoulou E., Giannimaras D.A., Sarno S., Simos G., Georgopoulou U., Mamalaki A. (2014). A complex signaling network involving protein kinase CK2 is required for hepatitis c virus core protein-mediated modulation of the iron-regulatory hepcidin gene expression. Cell. Mol. Life Sci..

[11] Meggio F., Marin O., Boschetti M., Sarno S., Pinna L.A. (2001). Hiv-1 rev transactivator: A beta-subunit directed substrate and effector of protein kinase CK2. Mol. Cell. Biochem..

[12] Pagano M.A., Arrigoni G., Marin O., Sarno S., Meggio F., Treharne K.J., Mehta A., Pinna L.A. (2008). Modulation of protein kinase CK2 activity by fragments of CFTR encompassing f508 may reflect functional links with cystic fibrosis pathogenesis. Biochemistry.

[13] Pagano M.A., Marin O., Cozza G., Sarno S., Meggio F., Treharne K.J., Mehta A., Pinna L.A. (2010). Cystic fibrosis transmembrane regulator fragments with the phe508 deletion exert a dual allosteric control over the master kinase CK2. Biochem. J..

[14] Cesaro L., Marin O., Venerando A., Donella-Deana A., Pinna L.A. (2013). Phosphorylation of cystic fibrosis transmembrane conductance regulator (CFTR) serine-511 by the combined action of tyrosine kinases and CK2: The implication of tyrosine-512 and phenylalanine-508. Amino Acids.

[15] Siddiqui-Jain A., Drygin D., Streiner N., Chua P., Pierre F., O’Brien S.E., Bliesath J., Omori M., Huser N., Ho C. (2010). Cx-4945, an orally bioavailable selective inhibitor of protein kinase CK2, inhibits prosurvival and angiogenic signaling and exhibits antitumor efficacy. Cancer Res..

[16] Pierre F., Chua P.C., O’Brien S.E., Siddiqui-Jain A., Bourbon P., Haddach M., Michaux J., Nagasawa J., Schwaebe M.K., Stefan E. (2011). Pre-clinical characterization of cx-4945, a potent and selective small molecule inhibitor of CK2 for the treatment of cancer. Mol. Cell. Biochem..

[17] Ferguson A.D., Sheth P.R., Basso A.D., Paliwal S., Gray K., Fischmann T.O., Le H.V. (2011). Structural basis of cx-4945 binding to human protein kinase CK2. FEBS Lett..

[18] Wilson L.K., Dhillon N., Thorner J., Martin G.S. (1997). Casein kinase ii catalyzes tyrosine phosphorylation of the yeast nucleolar immunophilin fpr3. J. Biol. Chem..

[19] Marin O., Meggio F., Sarno S., Cesaro L., Pagano M.A., Pinna L.A. (1999). Tyrosine versus serine/threonine phosphorylation by protein kinase casein kinase-2. A study with peptide substrates derived from immunophilin FPR3. J. Biol. Chem..

[20] Sarno S., Vaglio P., Meggio F., Issinger O.G., Pinna L.A. (1996). Protein kinase CK2 mutants defective in substrate recognition. Purification and kinetic analysis. J. Biol. Chem..

[21] Marin O., Sarno S., Boschetti M., Pagano M.A., Meggio F., Ciminale V., D’Agostino D.M., Pinna L.A. (2000). Unique features of hiv-1 rev protein phosphorylation by protein kinase CK2 ('casein kinase-2'). FEBS Lett..

[22] Poletto G., Vilardell J., Marin O., Pagano M.A., Cozza G., Sarno S., Falques A., Itarte E., Pinna L.A., Meggio F. (2008). The regulatory beta subunit of protein kinase CK2 contributes to the recognition of the substrate consensus sequence. A study with an EIF2 beta-derived peptide. Biochemistry.

[23] Meggio F., Boldyreff B., Marin O., Marchiori F., Perich J.W., Issinger O.G., Pinna L.A. (1992). The effect of polylysine on casein-kinase-2 activity is influenced by both the structure of the protein/peptide substrates and the subunit composition of the enzyme. Eur. J. Biochem..

[24] Boldyreff B., James P., Staudenmann W., Issinger O.G. (1993). Ser2 is the autophosphorylation site in the beta subunit from bicistronically expressed human casein kinase-2 and from native rat liver casein kinase-2 beta. Eur. J. Biochem..

[25] Litchfield D.W., Lozeman F.J., Cicirelli M.F., Harrylock M., Ericsson L.H., Piening C.J., Krebs E.G. (1991). Phosphorylation of the beta subunit of casein kinase ii in human a431 cells. Identification of the autophosphorylation site and a site phosphorylated by p34cdc2. J. Biol. Chem..

[26] Lolli G., Pinna L.A., Battistutta R. (2012). Structural determinants of protein kinase CK2 regulation by autoinhibitory polymerization. ACS Chem. Biol..

[27] Cozza G., Pinna L.A., Moro S. (2013). Kinase CK2 inhibition: An update. Curr. Med. Chem..

[28] Cozza G., Pinna L.A. (2015). Casein kinases as potential therapeutic targets. Expert Opin. Ther. Targets.

[29] Hubert A., Paris S., Piret J.P., Ninane N., Raes M., Michiels C. (2006). Casein Kinase 2 inhibition decreases hypoxia-inducible factor-1 activity under hypoxia through elevated p53 protein level. J. Cell Sci..

[30] Hupp T.R., Meek D.W., Midgley C.A., Lane D.P. (1992). Regulation of the specific DNA binding function of p53. Cell.

[31] Mottet D., Ruys S.P., Demazy C., Raes M., Michiels C. (2005). Role for casein kinase 2 in the regulation of HIF-1 activity. Int. J. Cancer.

[32] Pluemsampant S., Safronova O.S., Nakahama K., Morita I. (2008). Protein kinase CK2 is a key activator of histone deacetylase in hypoxia-associated tumors. Int. J. Cancer.

[33] Charo I.F., Taubman M.B. (2004). Chemokines in the pathogenesis of vascular disease. Circ. Res..

[34] Harvey E.J., Li N., Ramji D.P. (2007). Critical role for casein kinase 2 and phosphoinositide-3-kinase in the interferon-gamma-induced expression of monocyte chemoattractant protein-1 and other key genes implicated in atherosclerosis. Arter. Thromb. Vasc. Biol..

[35] Okochi M., Walter J., Koyama A., Nakajo S., Baba M., Iwatsubo T., Meijer L., Kahle P.J., Haass C. (2000). Constitutive phosphorylation of the parkinson's disease associated alpha-synuclein. J. Biol. Chem..

[36] Lee G., Tanaka M., Park K., Lee S.S., Kim Y.M., Junn E., Lee S.H., Mouradian M.M. (2004). Casein Kinase II-mediated phosphorylation regulates alpha-synuclein/synphilin-1 interaction and inclusion body formation. J. Biol. Chem..

[37] Ishii A., Nonaka T., Taniguchi S., Saito T., Arai T., Mann D., Iwatsubo T., Hisanaga S., Goedert M., Hasegawa M. (2007). Casein kinase 2 is the major enzyme in brain that phosphorylates ser129 of human alpha-synuclein: Implication for alpha-synucleinopathies. FEBS Lett..

[38] Iimoto D.S., Masliah E., DeTeresa R., Terry R.D., Saitoh T. (1990). Aberrant casein kinase II in alzheimer's disease. Brain Res..

[39] Aksenova M.V., Burbaeva G.S., Kandror K.V., Kapkov D.V., Stepanov A.S. (1991). The decreased level of casein kinase 2 in brain cortex of schizophrenic and alzheimer's disease patients. FEBS Lett..

[40] Masliah E., Iimoto D.S., Mallory M., Albright T., Hansen L., Saitoh T. (1992). Casein kinase ii alteration precedes tau accumulation in tangle formation. Am. J. Pathol..

[41] Greenwood J.A., Scott C.W., Spreen R.C., Caputo C.B., Johnson G.V. (1994). Casein kinase ii preferentially phosphorylates human tau isoforms containing an amino-terminal insert. Identification of threonine 39 as the primary phosphate acceptor. J. Biol. Chem..

[42] Yamada M., Katsuma S., Adachi T., Hirasawa A., Shiojima S., Kadowaki T., Okuno Y., Koshimizu T.A., Fujii S., Sekiya Y. (2005). Inhibition of protein kinase CK2 prevents the progression of glomerulonephritis. Proc. Natl. Acad. Sci. USA.

[43] Maekawa T., Kosuge S., Karino A., Okano T., Ito J., Munakata H., Ohtsuki K. (2000). Biochemical characterization of 60s acidic ribosomal p proteins from porcine liver and the inhibition of their immunocomplex formation with sera from systemic lupus erythematosus (sle) patients by glycyrrhizin in vitro. Biol. Pharm. Bull..

[44] Caponi L., Anzilotti C., Longombardo G., Migliorini P. (2007). Antibodies directed against ribosomal p proteins cross-react with phospholipids. Clin. Exp. Immunol..

[45] Hauck L., Harms C., An J., Rohne J., Gertz K., Dietz R., Endres M., von Harsdorf R. (2008). Protein kinase CK2 links extracellular growth factor signaling with the control of p27(kip1) stability in the heart. Nat. Med..

[46] Tapia J.C., Bolanos-Garcia V.M., Sayed M., Allende C.C., Allende J.E. (2004). Cell cycle regulatory protein p27kip1 is a substrate and interacts with the protein kinase CK2. J. Cell. Biochem..

[47] De Stefano D., Villella V.R., Esposito S., Tosco A., Sepe A., De Gregorio F., Salvadori L., Grassia R., Leone C.A., De Rosa G. (2014). Restoration of CFTR function in patients with cystic fibrosis carrying the f508del-CFTR mutation. Autophagy.

[48] Ruzzene M., Pinna L.A. (2010). Addiction to protein kinase CK2: A common denominator of diverse cancer cells?. Biochim. Biophys. Acta Proteins Proteom..

[49] Cozza G., Mazzorana M., Papinutto E., Bain J., Elliott M., di Maira G., Gianoncelli A., Pagano M.A., Sarno S., Ruzzene M. (2009). Quinalizarin as a potent, selective and cell-permeable inhibitor of protein kinase CK2. Biochem. J..

[50] Cozza G., Bonvini P., Zorzi E., Poletto G., Pagano M.A., Sarno S., Donella-Deana A., Zagotto G., Rosolen A., Pinna L.A. (2006). Identification of ellagic acid as potent inhibitor of protein kinase CK2: A successful example of a virtual screening application. J. Med. Chem..

[51] Wang J., Wolf R.M., Caldwell J.W., Kollman P.A., Case D.A. (2004). Development and testing of a general amber force field. J. Comput. Chem..

[52] Brooks B.R., Brooks C.L., Mackerell A.D., Nilsson L., Petrella R.J., Roux B., Won Y., Archontis G., Bartels C., Boresch S. (2009). Charmm: The biomolecular simulation program. J. Comput. Chem..

[53] Halgren T.A. (1996). Merck molecular force field. 1. Basis, form, scope, parameterization, and performance of mmff94. J. Comput. Chem..

[54] Harder E., Damm W., Maple J., Wu C.J., Reboul M., Xiang J.Y., Wang L.L., Lupyan D., Dahlgren M.K., Knight J.L. (2016). Opls3: A force field providing broad coverage of drug-like small molecules and proteins. J. Chem. Theory Comput..

[55] Cozza G., Gianoncelli A., Bonvini P., Zorzi E., Pasquale R., Rosolen A., Pinna L.A., Meggio F., Zagotto G., Moro S. (2011). Urolithin as a converging scaffold linking ellagic acid and coumarin analogues: Design of potent protein kinase CK2 inhibitors. ChemMedChem.

[56] Sarno S., Moro S., Meggio F., Zagotto G., Dal Ben D., Ghisellini P., Battistutta R., Zanotti G., Pinna L.A. (2002). Toward the rational design of protein kinase casein kinase-2 inhibitors. Pharmacol. Ther..

[57] Pagano M.A., Sarno S., Poletto G., Cozza G., Pinna L.A., Meggio F. (2005). Autophosphorylation at the regulatory beta subunit reflects the supramolecular organization of protein kinase CK2. Mol. Cell. Biochem..

[58] Kroemer R.T. (2007). Structure-based drug design: Docking and scoring. Curr. Protein Pept. Sci..

[59] Cavasotto C.N., Orry A.J. (2007). Ligand docking and structure-based virtual screening in drug discovery. Curr. Top. Med. Chem..

[60] Morris G.M., Huey R., Lindstrom W., Sanner M.F., Belew R.K., Goodsell D.S., Olson A.J. (2009). Autodock4 and autodocktools4: Automated docking with selective receptor flexibility. J. Comput. Chem..

[61] Jones G., Willett P., Glen R.C., Leach A.R., Taylor R. (1997). Development and validation of a genetic algorithm for flexible docking. J. Mol. Biol..

[62] Vilar S., Cozza G., Moro S. (2008). Medicinal chemistry and the molecular operating environment (moe): Application of qsar and molecular docking to drug discovery. Curr. Top. Med. Chem..

[63] Allen W.J., Balius T.E., Mukherjee S., Brozell S.R., Moustakas D.T., Lang P.T., Case D.A., Kuntz I.D., Rizzo R.C. (2015). Dock 6: Impact of new features and current docking performance. J. Comput. Chem..

[64] Friesner R.A., Banks J.L., Murphy R.B., Halgren T.A., Klicic J.J., Mainz D.T., Repasky M.P., Knoll E.H., Shelley M., Perry J.K. (2004). Glide: A new approach for rapid, accurate docking and scoring. 1. Method and assessment of docking accuracy. J. Med. Chem..

[65] McGann M. (2011). Fred pose prediction and virtual screening accuracy. J. Chem. Inf. Model..

[66] Zsoldos Z., Reid D., Simon A., Sadjad S.B., Johnson A.P. (2007). Ehits: A new fast, exhaustive flexible ligand docking system. J. Mol. Graph. Model..

[67] Rarey M., Kramer B., Lengauer T., Klebe G. (1996). A fast flexible docking method using an incremental construction algorithm. J. Mol. Biol..

[68] Bain J., Plater L., Elliott M., Shpiro N., Hastie C.J., McLauchlan H., Klevernic I., Arthur J.S., Alessi D.R., Cohen P. (2007). The selectivity of protein kinase inhibitors: A further update. Biochem. J..

[69] De Moliner E., Moro S., Sarno S., Zagotto G., Zanotti G., Pinna L.A., Battistutta R. (2003). Inhibition of protein kinase CK2 by anthraquinone-related compounds. A structural insight. J. Biol. Chem..

[70] Cozza G., Venerando A., Sarno S., Pinna L.A. (2015). The selectivity of CK2 inhibitor quinalizarin: A reevaluation. Biomed. Res. Int..

[71] Franchin C., Salvi M., Arrigoni G., Pinna L.A. (2015). Proteomics perturbations promoted by the protein kinase CK2 inhibitor quinalizarin. Biochim. Biophys. Acta.

[72] Vangrevelinghe E., Zimmermann K., Schoepfer J., Portmann R., Fabbro D., Furet P. (2003). Discovery of a potent and selective protein kinase CK2 inhibitor by high-throughput docking. J. Med. Chem..

[73] Sarno S., de Moliner E., Ruzzene M., Pagano M.A., Battistutta R., Bain J., Fabbro D., Schoepfer J., Elliott M., Furet P. (2003). Biochemical and three-dimensional-structural study of the specific inhibition of protein kinase CK2 by [5-oxo-5,6-dihydroindolo-(1,2-a)quinazolin-7-yl]acetic acid (iqa). Biochem. J..

[74] Sun H., Xu X., Wu X., Zhang X., Liu F., Jia J., Guo X., Huang J., Jiang Z., Feng T. (2013). Discovery and design of tricyclic scaffolds as protein kinase CK2 (CK2) inhibitors through a combination of shape-based virtual screening and structure-based molecular modification. J. Chem. Inf. Model..

[75] Sun H., Wu X., Xu X., Jiang Z., Liu Z., You Q. (2014). Discovery of novel CK2 leads by cross-docking based virtual screening. Med. Chem..

[76] Genheden S., Ryde U. (2015). The mm/pbsa and mm/gbsa methods to estimate ligand-binding affinities. Expert Opin. Drug Discov..

[77] Carlsson J., Boukharta L., Aqvist J. (2008). Combining docking, molecular dynamics and the linear interaction energy method to predict binding modes and affinities for non-nucleoside inhibitors to hiv-1 reverse transcriptase. J. Med. Chem..

[78] Sekiguchi Y., Nakaniwa T., Kinoshita T., Nakanishi I., Kitaura K., Hirasawa A., Tsujimoto G., Tada T. (2009). Structural insight into human CK2alpha in complex with the potent inhibitor ellagic acid. Bioorg. Med. Chem. Lett..

[79] Chilin A., Battistutta R., Bortolato A., Cozza G., Zanatta S., Poletto G., Mazzorana M., Zagotto G., Uriarte E., Guiotto A. (2008). Coumarin as attractive casein kinase 2 (CK2) inhibitor scaffold: An integrate approach to elucidate the putative binding motif and explain structure-activity relationships. J. Med. Chem..

[80] Zandomeni R., Zandomeni M.C., Shugar D., Weinmann R. (1986). Casein kinase type ii is involved in the inhibition by 5,6-dichloro-1-beta-d-ribofuranosylbenzimidazole of specific rna polymerase ii transcription. J. Biol. Chem..

[81] Ruzzene M., Penzo D., Pinna L.A. (2002). Protein kinase CK2 inhibitor 4,5,6,7-tetrabromobenzotriazole (TBB) induces apoptosis and caspase-dependent degradation of haematopoietic lineage cell-specific protein 1 (hs1) in jurkat cells. Biochem. J..

[82] Pagano M.A., Meggio F., Ruzzene M., Andrzejewska M., Kazimierczuk Z., Pinna L.A. (2004). 2-dimethylamino-4,5,6,7-tetrabromo-1h-benzimidazole: A novel powerful and selective inhibitor of protein kinase CK2. Biochem. Biophys. Res. Commun..

[83] Gianoncelli A., Cozza G., Orzeszko A., Meggio F., Kazimierczuk Z., Pinna L.A. (2009). Tetraiodobenzimidazoles are potent inhibitors of protein kinase CK2. Bioorg. Med. Chem..

[84] Cozza G., Girardi C., Ranchio A., Lolli G., Sarno S., Orzeszko A., Kazimierczuk Z., Battistutta R., Ruzzene M., Pinna L.A. (2014). Cell-permeable dual inhibitors of protein kinases CK2 and pim-1: Structural features and pharmacological potential. Cell. Mol. Life Sci..

[85] Lorusso P.M., Eder J.P. (2008). Therapeutic potential of novel selective-spectrum kinase inhibitors in oncology. Expert Opin. Investig. Drugs.

[86] Lackey K.E. (2006). Lessons from the drug discovery of lapatinib, a dual erbb1/2 tyrosine kinase inhibitor. Curr. Top. Med. Chem..

[87] Zhang Y.M., Cockerill S., Guntrip S.B., Rusnak D., Smith K., Vanderwall D., Wood E., Lackey K. (2004). Synthesis and sar of potent egfr/erbb2 dual inhibitors. Bioorg. Med. Chem. Lett..

[88] Brault L., Gasser C., Bracher F., Huber K., Knapp S., Schwaller J. (2010). Pim serine/threonine kinases in the pathogenesis and therapy of hematologic malignancies and solid cancers. Haematologica.

[89] Cozza G., Zanin S., Sarno S., Costa E., Girardi C., Ribaudo G., Salvi M., Zagotto G., Ruzzene M., Pinna L.A. (2015). Design, validation and efficacy of bisubstrate inhibitors specifically affecting ecto-CK2 kinase activity. Biochem. J..

[90] Kozakov D., Brenke R., Comeau S.R., Vajda S. (2006). Piper: An fft-based protein docking program with pairwise potentials. Proteins.

[91] Phillips J.C., Braun R., Wang W., Gumbart J., Tajkhorshid E., Villa E., Chipot C., Skeel R.D., Kale L., Schulten K. (2005). Scalable molecular dynamics with namd. J. Comput. Chem..

[92] Golub A.G., Bdzhola V.G., Briukhovetska N.V., Balanda A.O., Kukharenko O.P., Kotey I.M., Ostrynska O.V., Yarmoluk S.M. (2011). Synthesis and biological evaluation of substituted (thieno[2,3-d]pyrimidin-4-ylthio)carboxylic acids as inhibitors of human protein kinase CK2. Eur. J. Med. Chem..

[93] Ostrynska O.V., Balanda A.O., Bdzhola V.G., Golub A.G., Kotey I.M., Kukharenko O.P., Gryshchenko A.A., Briukhovetska N.V., Yarmoluk S.M. (2016). Design and synthesis of novel protein kinase CK2 inhibitors on the base of 4-aminothieno[2,3-d]pyrimidines. Eur. J. Med. Chem..

[94] Borowiecki P., Wawro A.M., Winska P., Wielechowska M., Bretner M. (2014). Synthesis of novel chiral tbbt derivatives with hydroxyl moiety. Studies on inhibition of human protein kinase CK2alpha and cytotoxicity properties. Eur. J. Med. Chem..

[95] Hochscherf J., Lindenblatt D., Steinkruger M., Yoo E., Ulucan O., Herzig S., Issinger O.G., Helms V., Gotz C., Neundorf I. (2015). Development of a high-throughput screening-compatible assay to identify inhibitors of the CK2alpha/CK2beta interaction. Anal. Biochem..

[96] Zhou Y., Li X., Zhang N., Zhong R. (2015). Structural basis for low-affinity binding of non-r2 carboxylate-substituted tricyclic quinoline analogs to CK2alpha: Comparative molecular dynamics simulation studies. Chem. Biol. Drug Des..

[97] Retegan M., Milet A., Jamet H. (2009). Exploring the binding of inhibitors derived from tetrabromobenzimidazole to the CK2 protein using a qm/mm-pb/sa approach. J. Chem. Inf. Model..

[98] Langer T., Hoffmann R.D. (2006). Pharmacophores and pharmacophore searches.

[99] Chirico N., Gramatica P. (2012). Real external predictivity of qsar models. Part 2. New intercomparable thresholds for different validation criteria and the need for scatter plot inspection. J. Chem. Inf. Model..

[100] Chirico N., Gramatica P. (2011). Real external predictivity of qsar models: How to evaluate it? Comparison of different validation criteria and proposal of using the concordance correlation coefficient. J. Chem. Inf. Model..

[101] Di-wu L., Li L.L., Wang W.J., Xie H.Z., Yang J., Zhang C.H., Huang Q., Zhong L., Feng S., Yang S.Y. (2012). Identification of CK2 inhibitors with new scaffolds by a hybrid virtual screening approach based on bayesian model; pharmacophore hypothesis and molecular docking. J. Mol. Graph. Model..

[102] Srivastava R., Akthar S., Sharma R., Mishra S. (2015). Identification of ellagic acid analogues as potent inhibitor of protein kinase CK2:A chemopreventive role in oral cancer. Bioinformation.

[103] Morshed M.N., Muddassar M., Pasha F.A., Cho S.J. (2009). Pharmacophore identification and validation study of CK2 inhibitors using comfa/comsia. Chem. Biol. Drug Des..

[104] Zhang N., Zhong R. (2010). Docking and 3d-qsar studies of 7-hydroxycoumarin derivatives as CK2 inhibitors. Eur J. Med. Chem..

