# Supplementary material for: The Development of CK2 Inhibitors: From Traditional Pharmacology to in Silico Rational Drug Design
Source: Pharmaceuticals (Basel). 2017 Feb 20;10(1):26. doi: 10.3390/ph10010026 (PMC5374430; doi:10.3390/ph10010026)
Supplement: Supplementary file 1 [file pharmaceuticals-10-00026-s001.pdf]

Supplementary Materials: The Development of CK2 Inhibitors: From Traditional Pharmacology to in Silico Rational Drug Design

Giorgio Cozza

Table S1. Summary of all CK2 crystal structures deposited in the Protein Data Bank (PDB).

PDB ID	Organism	Ligand	Resolution (Å)	Year
1DAW	*Zea mays*	ANP	2.20	2000
1DAY	*Zea mays*	GNP	2.20	2000
1DS5	*Zea mays*	AMP	3.16	2001
1F0Q	*Zea mays*	Emodin	2.63	2001
1J91	*Zea mays*	TBB	2.22	2002
1JAM	*Zea mays*	------	2.18	2002
1JWH	*Homo sapiens*	ANP	3.10	2002
1LP4	*Zea mays*	ANP	1.86	2002
1LR4	*Zea mays*	BEN	2.00	2002
1LPU	*Zea mays*	BEN	1.86	2002
1M2P	*Zea mays*	HNA	2.00	2003
1M2R	*Zea mays*	MNY	1.70	2003
1M2Q	*Zea mays*	MNX	1.79	2003
1NA7	*Homo sapiens*	------	2.40	2003
1PJK	*Homo sapiens*	ANP	2.50	2003
1OM1	*Zea mays*	IQA	1.68	2004
1ZOH	*Zea mays*	K44	1.81	2005
1ZOG	*Zea mays*	K37	2.30	2005
1ZOE	*Zea mays*	K25	1.77	2005
2OXX	*Zea mays*	K22	2.30	2007
2OXD	*Zea mays*	K32	2.30	2007
2OXY	*Zea mays*	K17	1.81	2007
2PVR	*Homo sapiens*	ANP	1.61	2007
2PVJ	*Zea mays*	P44	1.70	2008
2PVM	*Zea mays*	P29	2.00	2008
2PVH	*Zea mays*	P19	2.20	2008
2PVK	*Zea mays*	P45	1.90	2008
2PVL	*Zea mays*	P55	1.90	2008
2PVN	*Zea mays*	P63	2.00	2008
2QC6	*Zea mays*	DBC	1.85	2008
2R7I	*R. norvegicus*	------	3.00	2008
3BE9	*Zea mays*	P04	2.00	2008
3C13	*Homo sapiens*	Emodin	1.95	2008
3BQC	*Homo sapiens*	Emodin	1.50	2008
3FL5	*Zea mays*	Quinalizarin	2.30	2009
3FWQ	*Homo Sapiens*	glycerol	3.20	2009
3H30	*Homo sapiens*	DRB	1.56	2009
3E2B	*Homo sapiens (α′)*	CCK	3.20	2009
3JUH	*Homo sapiens*	ANP	1.66	2009

2ZJW	*Homo sapiens*	Ellagic acid	2.40	2009
3KXM	*Zea mays*	K74	1.75	2010
3KXN	*Zea mays*	K88	2.00	2010
3KXH	*Zea mays*	K66	1.70	2010
3KXG	*Zea mays*	K64	1.70	2010
3PVG	*Zea mays*	K68	1.50	2010
3NGA	*Homo sapiens*	CX-4945	2.71	2010
3NSZ	*Homo sapiens*	ANP	1.30	2010
3OWJ	*Homo sapiens*	1EL	1.85	2010
3OWK	*Homo sapeins*	18E	1.80	2010
3OWL	*Homo sapiens*	19E	2.10	2010
3MB6	*Homo sapiens*	01I	1.75	2010
3MB7	*Homo sapiens*	14I	1.65	2010
3PWD	*Zea mays*	NBC	2.20	2011
3PZH	*Zea mays*	Emodin	1.92	2011
3Q04	*Homo sapiens*	------	1.80	2011
3AT2	*Homo sapiens*	------	1.60	2011
3AMY	*Homo sapiens*	Apigenin	2.30	2011
3RP0	*Homo sapiens*	ANP	3.00	2011
3ROT	*Homo sapiens*	CX-5279	1.75	2011
1F0Q	*Zea mays*	Emodin	2.63	2001
1J91	*Zea mays*	TBB	2.22	2002
1JAM	*Zea mays*	------	2.18	2002
1JWH	*Homo sapiens*	ANP	3.10	2002
1LP4	*Zea mays*	ANP	1.86	2002
1LR4	*Zea mays*	BEN	2.00	2002
1LPU	*Zea mays*	BEN	1.86	2002
1M2P	*Zea mays*	HNA	2.00	2003
1M2R	*Zea mays*	MNY	1.70	2003
1M2Q	*Zea mays*	MNX	1.79	2003
1NA7	*Homo sapiens*	------	2.40	2003
1PJK	*Homo sapiens*	ANP	2.50	2003
1OM1	*Zea mays*	IQA	1.68	2004
1ZOH	*Zea mays*	K44	1.81	2005
1ZOG	*Zea mays*	K37	2.30	2005
1ZOE	*Zea mays*	K25	1.77	2005
2OXX	*Zea mays*	K22	2.30	2007
2OXD	*Zea mays*	K32	2.30	2007
2OXY	*Zea mays*	K17	1.81	2007
2PVR	*Homo sapiens*	ANP	1.61	2007
2PVJ	*Zea mays*	P44	1.70	2008
2PVM	*Zea mays*	P29	2.00	2008
2PVH	*Zea mays*	P19	2.20	2008
2PVK	*Zea mays*	P45	1.90	2008
2PVL	*Zea mays*	P55	1.90	2008
2PVN	*Zea mays*	P63	2.00	2008
2QC6	*Zea mays*	DBC	1.85	2008
2R7I	*R. norvegicus*	------	3.00	2008
3BE9	*Zea mays*	P04	2.00	2008
3C13	*Homo sapiens*	Emodin	1.95	2008
3BQC	*Homo sapiens*	Emodin	1.50	2008
3FL5	*Zea mays*	Quinalizari n	2.30	2009

3FWQ	*Homo Sapiens*	glycerol	3.20	2009
3H30	*Homo sapiens*	DRB	1.56	2009
3E2B	*Homo sapiens (α′)*	CCK	3.20	2009
3JUH	*Homo sapiens*	ANP	1.66	2009
2ZJW	*Homo sapiens*	Ellagic acid	2.40	2009
3KXM	*Zea mays*	K74	1.75	2010
3KXN	*Zea mays*	K88	2.00	2010
3KXH	*Zea mays*	K66	1.70	2010
3KXG	*Zea mays*	K64	1.70	2010
3PVG	*Zea mays*	K68	1.50	2010
3NGA	*Homo sapiens*	CX-4945	2.71	2010
3NSZ	*Homo sapiens*	ANP	1.30	2010
3OWJ	*Homo sapiens*	1EL	1.85	2010
3OWK	*Homo sapeins*	18E	1.80	2010
3OWL	*Homo sapiens*	19E	2.10	2010
3MB6	*Homo sapiens*	01I	1.75	2010
3MB7	*Homo sapiens*	14I	1.65	2010
3PWD	*Zea mays*	NBC	2.20	2011
3PZH	*Zea mays*	Emodin	1.92	2011
3Q04	*Homo sapiens*	------	1.80	2011
3AT2	*Homo sapiens*	------	1.60	2011
3AMY	*Homo sapiens*	Apigenin	2.30	2011
3RP0	*Homo sapiens*	ANP	3.00	2011
3ROT	*Homo sapiens*	CX-5279	1.75	2011
3PE1	*Homo sapiens*	CX-4945	1.60	2011
3PE2	*Homo sapiens*	CX-5011	1.90	2011
3OFM	*Homo sapiens (α′)*	4B0	2.00	2011
3AT3	*Homo sapiens*	ATK	2.60	2011
3AT4	*Homo sapiens*	CCK	2.20	2011
3RPS	*Homo sapiens*	4B0	2.30	2011
3Q9W	*Homo sapiens*	Emodin	1.70	2012
3Q9Y	*Homo sapiens*	Quinalizari n	1.80	2012
3Q9X	*Homo sapiens*	Emodin	2.20	2012
3Q9Z	*Homo sapiens*	Quinalizari n	2.20	2012
3AXW	*Homo sapiens*	TID	2.50	2012
3U9C	*Homo sapiens*	Resorufin	3.20	2012
4DGO	*Zea mays*	0JW	1.96	2012
4DGM	*Zea mays*	Apigenin	1.65	2012
4DGN	*Zea mays*	Luteolin	1.75	2012
4DGL	*Homo sapiens*	------	-----	2012
4ANM	*Zea mays*	WUL	1.70	2012
3QA0	*Homo sapiens*	------	2.50	2012
3U4U	*Homo sapiens*	LNH	2.20	2012
4FBX	*Homo sapiens*	0TJ	2.33	2012
3U87	*Homo sapiens*	ANP	2.90	2012
3W8L	*Homo sapiens*	I6P	2.40	2013
3WAR	*Homo sapiens*	-----	1.04	2013
4IB5	*Homo sapiens*	CL	2.20	2013
4FI1	*S. cerevisiae*	ATP	2.09	2013
4IB5	*Homo sapiens*	Pc (peptide)	2.20	2013
4GRB	*Homo sapiens*	0XG	2.15	2013

4GUB	*Homo sapiens*	0Y4	2.20	2013
4MWH	*S. cerevisiae*	ATP	2.09	2013
4MD7	*Homo sapiens*	-----	3.10	2013
4MD8	*Homo sapiens*	-----	3.30	2013
4MD9	*Homo sapiens*	-----	3.50	2013
4NH1	*Homo sapiens*	-----	3.30	2014
4JQE	*S. cerevisiae*	ANP	1.77	2014
4JR7	*S. cerevisiae*	GNP	1.48	2014
4LFI	*S. cerevisiae*	GNP	1.85	2014
4KWP	*Homo sapiens*	TDB	1.25	2014
4BXA	*Homo sapiens*	JRJ	2.65	2014
4BXB	*Homo sapiens*	JRJ	2.35	2014
3WIK	*Homo sapiens*	LCD	2.00	2014
3WIL	*Homo sapiens*	LCT	2.90	2014
4RLK	*Zea mays*	E91	1.24	2015
4RLL	*Zea mays*	E91	1.85	2015
3WOW	*Homo sapiens*	ANP	2.50	2015
4UB7	*Homo sapiens*	FLC26	2.21	2015
4UBA	*Homo sapiens*	FLC26	2.99	2015
5CQU	*Homo sapiens*	JRJ	2.35	2015
5CQW	*Homo sapiens*	JRJ	2.65	2015
3WIL	*Homo sapiens*	Comp. 3	2.90	2014
5B0X	*Homo sapiens*	HCK	2.30	2016
5H8B	*Homo sapiens*	5Y2	2.55	2016
5H8E	*Homo sapiens*	5Y3	2.15	2016
5H8G	*Homo sapiens*	5Y4	2.00	2016
5CLP	*Homo sapiens*	42J	1.68	2016
5CS6	*Homo sapiens*	K82	1.88	2016
5CSH	*Homo sapiens*	54E	1.59	2016
5CSP	*Homo sapiens*	54G	1.50	2016
5CSV	*Homo sapiens*	GAB	1.38	2016
5CU3	*Homo sapiens*	54S	1.79	2016
5CU4	*Homo sapiens*	54S	1.56	2016
5CU6	*Homo sapiens*	ATP	1.36	2016
5CVF	*Homo sapiens*	54Z	1.63	2016
5CVG	*Homo sapiens*	-----	1.25	2016
5CVH	*Homo sapiens*	ADP	1.85	2016
5CT0	*Homo sapiens*	54P	2.01	2016
5CTP	*Homo sapiens*	54R	2.03	2016
5CU0	*Homo sapiens*	54G	2.18	2016
5CU2	*Homo sapiens*	54G, 551	1.71	2016
5CX9	*Homo sapiens*	551	1.73	2016
